# The Story of the Insane from Year to Year

**Published:** 1895-12-21

**Authors:** 


### THE STORY OF THE INSANE FROM YEAR TO YEAR.

Eighth Series.

### THE WHITTINGHAM ASYLUM, LANCASHIRE.

Here there was an increase of 65 patients during the year, making a total of 1,930. The admissions reached 432, which means that the medical superintendent had the cases of 432 fresh patients to keep in his mind, in addition to many acute cases among the admissions of the previous year, and that in addition to those absurd "continuation orders" he had to prepare and sign 432 certificates of the mental and bodily state of these admissions. There were 118 recoveries and 229 deaths. The latter shows the medical superintendent would have to prepare and sign 1,374 certificates of death. Thrown into percentages these numbers show that the recoveries work out at 27·31 per cent. of the admissions, and the deaths at 12·05 per cent. on the average number resident. The recovery rate is below the average, and the death rate is above it. Of the deaths 88 were caused by cerebral and spinal diseases, 31 by pneumonia, 34 by consumption, and 9 by dysentery. Hereditary tendency caused the insanity in 136 of the admissions, intemperance in drink in 120, religion in 9, and love in 11. Dr. Perceval remarks on the number of cases owing their insanity to hereditary tendency, and he hopes that "as the people become better informed, and recognise the risk they run of imperilling their life's happiness by marrying into insane stock, the number of these cases may diminish." We hope so also, but we fear Dr. Perceval hardly realises the carelessness and denseness of the British public in these matters. For the last eight years this journal has pointed out this risk, or rather this certainty—this criminality of marriage where the tendency to insanity exists; but we should be only too glad to hear of a few cases in which our words have acted as deterrents. As already stated, nine of the deaths were from dysentery. The drainage seems to be in a bad state, for we are told that "drains run underneath wards and corridors; the down-spouts carrying roof water run direct into drains. They are not trapped, and act, in fact, as ventilators." At the annexe, which is only a few years old, the drain-pipes "are not cemented, but merely puddled; large cesspools exist close to the wards, and these are not properly disconnected." When referring to Dr. Wallis' resignation, the visitors "desire to take this opportunity of placing on record their high appreciation of the services rendered to the county and the asylum by Dr. Wallis." There was a balance of £555 in favour of the farm after estimating rent of land, and including rates and taxes. The average weekly cost per head was a fraction under 8s. 4d.

### THE PRESTWICH ASYLUM, LANCASHIRE.

This is, we believe, the largest asylum in the world. It contained at the close of 1894 almost 2,500 patients. There were 687 admissions, 304 recoveries, and 243 deaths. No argument, beyond quoting these numbers, is necessary to convince everyone what mistakes these large asylums are. The percentage of recoveries on admissions is 44·63; that of the deaths on the average number resident is 9·81. As usual, the two most potent causes of insanity in those admitted are drink and hereditary influence, the former accounting for 130, and the latter fo 137. Of the deaths 102 were caused by cerebral and spinal diseases, and 61 by consumption. The pressure on the accommodation which was noted in the report for 1893 continued during 1894, and, as Mr. Ley expresses it, this overcrowding "constituted an overtax on its resources and subjected it to a strain both inconvenient and undesirable." Now, an asylum of any size, much less an enormous one like Prestwich, has no need of an increase to the burden which it entails on the staff, and yet it is strange to notice how almost invariably the managers postpone building for the necessary requirements of their insane poor. This postponement adds immensely to the worry and responsibility of everyone having charge of lunatics, and it increases the cost to the ratepayer, for very often room must be found either in other counties or in licensed houses. Lancashire, to keep pace with the numbers of its registered insane, should build an asylum every third or fourth year. When speaking of the staff of attendants and nurses Mr. Ley says no effort should be spared to reduce the causes of unrest and dissatisfaction which have made their appearance, and to get rid of these he rightly puts the establishment of a system of pensions in the first rank for eradicating these feelings of dissatisfaction; and he adds, "The uncertainty of the existing procedure is tending to bring asylum service in Lancashire into disrepute, and is causing much heartburning among the older officials." The weekly charge is 9s. 4d.

### DUNDEE ROYAL ASYLUM.

At the close of the year this asylum contained 406 patients. There were 163 admissions, 51 recoveries, and 55 deaths. The recoveries stand at 31·28 per cent. of the admissions, and the deaths at 13·21 per cent. on the average number resident. Twenty of the deaths were owing to brain disease and five to consumption. Intemperance in drink caused the insanity in 37 of the admissions, and hereditary influence in 15. All our public asylums contain a few patients whom it is very difficult to classify. They are very troublesome, and never blend well with the rest of the inmates. Dr. Rorie thinks this class—well enough known to all asylum medical officers—"ought probably to be classed with the habitual offenders, and generally are admitted from the prison or from the police office. Although probably too weak-minded to be suitable for prison discipline, they are certainly in many instances too sane for detention and treatment in an ordinary asylum. They refuse to engage in any form of occupation, declaring they had not been sent to the asylum for that purpose. They frequently attempt to pick the door-locks, but the most striking feature is that they combine in these efforts a procedure of very rare occurrence amongst the ordinary insane." There is no doubt that these cases form a class by themselves, and ought not to be sent to our county asylums. This has been pointed out again and again, and we believe it has been brought under notice of Government, but not being a party question nothing has been done, nor is anything likely to be done, although it is surely bad enough to be insane without being compelled to associate with criminals. At this asylum private patients are received at rates varying from £25 to £180 a year. Paupers are charged from 11s. to 12s. 6d. a week.

### ARGYLE AND BUTE ASYLUM.

This asylum is situated at Lochgilphead. On the first of January, 1894, it contained 386 patients, and by the end of the year the number had risen to 408. There were 91 admissions, 27 recoveries, 16 discharged relieved, and 13 deaths. The percentages are given on two tables—one applicable to Argyle, and the other to Bute. For Argyle the percentage of recoveries is given as 27 calculated on the admissions, and the deaths as 16 per cent. on the average number resident; but this must be a mistake, as there were 322 Argyle patients and only 11 deaths. Only one death was due to brain disease. Six were caused by thoracic affections, and one by enteritis. Intemperance in drink caused the insanity in four of the admissions, domestic trouble and religious excitement in 5, and hereditary influence in 14. During the year 17 patients escaped. This would be considered a very high proportion in English asylums; but the frequency may be owing to an increased amount of liberty, and if so the liberty is cheaply bought. Sixteen of Dr. Cameron's attendants and nurses have obtained the certificate of proficiency in mental nursing, and others are being prepared for the examinations by the assistant medical officer. The Commissioners say that "great ability is shown both in the medical treatment of the patients and in the general management of the establishment." The weekly cost of maintenance stands at 8s. 6¾d.

